# Then and Now

**Published:** 1889-04

**Authors:** 


### THEN AND NOW.

We commend the following extract from a letter of John Henshall, late Christian Minister, published in the *Golden Gate* of March 15th, to such of our orthodox readers, as cling to the letter of "holy writ," but deny its spirit.

"I have often wondered of late, that during my life as an orthodox minister, it never occurred to me that my disbelief in ghost stories was in fact a disbelief in the Bible—that the most startling of them found in any book whether of fiction or fact, or related by any friend, is equaled by narratives in the Bible—indeed, that if every tale of supernatural appearance told in our day, every tale of midnight silence broken by some mysterious voice, or chamber solitude invaded by some flitting figure, whose pale and shadowy form caused the spectator's hair to stand up, every tale of the materialization of a spectral being through the intervention of a professed "medium," were a *pure invention*, it is at least copied after stories in the Bible. If the disciples of orthodoxy argued about the apparitions of the Bible according to their common methods of reasoning, they would say: "I know that spirits appear to men; it matters not whether any human book records the fact or not, or whether any living person testifies to it or not, the Bible says it; in short the deductions of reason, or the revelations of science, or the experiences of men are lighter than a feather, either as being in conflict with, or confirmatory of, anything that the Bible says."

"But many of them seem incapable of so reasoning in regard to apparitions. They are possessed of the notion that when their friends die, they ascend as they say Christ did, to some place called heaven, leaving platform above platform of worlds far beneath their feet, to say nothing of poor planet earth. But I repeat it, the Bible goes on the assumption of the continued communication of spirits with men; it goes on the assumption that they are around us, and may assume human shape, and suddenly become visible and vocal."

